# Case report series: Pembrolizumab and enfortumab vedotin in plasmacytoid urothelial carcinoma

**DOI:** 10.3389/fonc.2025.1608291

**Published:** 2025-09-08

**Authors:** Susanna G. Bowen, Derek B. Allison, Patrick J. Hensley, Zin W. Myint

**Affiliations:** ^1^ College of Medicine, University of Kentucky, Lexington, KY, United States; ^2^ Department of Pathology and Laboratory Medicine, University of Kentucky, Lexington, KY, United States; ^3^ Department of Urology, University of Kentucky, Lexington, KY, United States; ^4^ Markey Cancer Center, University of Kentucky, Lexington, KY, United States; ^5^ Department of Medicine, Division of Medical Oncology, University of Kentucky, Lexington, KY, United States

**Keywords:** plasmacytoid, urothelial carcinoma, immune-checkpoint inhibitors, antibody drug conjugate, pembrolizumab, enfortumab vedotin

## Abstract

Plasmacytoid urothelial carcinoma (PUC) is a rare and aggressive histologic subtype of urothelial carcinoma with no well-established treatment. Recently, the combination of pembrolizumab and enfortumab vedotin has become the standard of care for locally advanced and metastatic urothelial carcinoma due to improved survival outcomes in the EV-302 trial, but the number of histological subtypes in this trial is unknown. This case series presents three patients with Stage IV PUC who were treated with the combination of pembrolizumab and enfortumab vedotin. Two of the three patients demonstrated sustained stable disease after eight and ten months of treatment with this combination with manageable adverse effects including rash and colitis. The third patient experienced disease progression to leptomeningeal involvement eight months following initial diagnosis and subsequently succumbed to the disease. These observations support the potential efficacy of pembrolizumab in combination with enfortumab vedotin as a therapeutic option for this aggressive urothelial carcinoma subtype.

## 1 Introduction

Plasmacytoid urothelial carcinoma (PUC) is a rare and aggressive histologic subtype of urothelial carcinoma, accounting for only 1-3% of cases, whereas conventional urothelial carcinoma comprises approximately 80% (1). Histologically, PUC is characterized by discohesive cells, frequently invasive along deep tissue planes, with eccentrically placed nuclei and abundant eosinophilic cytoplasm, often accompanied by signet ring cell morphology (2–5). Clinically, PUC typically presents at an advanced stage, with symptoms such as gross hematuria and a high propensity for lymph node metastasis and peritoneal spread (3, 6–8). Due to its aggressive nature and late presentation, PUC is associated with poorer survival outcomes compared to urothelial carcinoma of other histologic subtypes (3, 6).

Due to the rarity of PUC, there is no well-established standard of care treatment strategy. However, systemic cisplatin-based combination chemotherapy with radical cystectomy is the most studied approach for resectable cases (2, 6, 9, 10). The survival rate to chemotherapy in urothelial carcinoma is historically very low with a 5-year survival rate <5%, and many patients are ineligible to receive cisplatin-based chemotherapy due to pre-existing comorbidities (7, 9, 11–13). Given the need for more effective treatment options, pembrolizumab was introduced into the treatment paradigm after KEYNOTE-045 demonstrated its efficacy in urothelial carcinoma when compared to chemotherapy (14). Additionally, the KEYNOTE-052 trial showed overall survival benefits in cisplatin-ineligible patients (9) leading to FDA approval of pembrolizumab in advanced urothelial carcinoma (15). After the EV-201 trial, enfortumab vedotin was approved as a third line treatment for locally advanced or metastatic urothelial carcinoma after pembrolizumab and chemotherapy (16). More recently, the EV-302 trial showed significantly improved survival outcomes with the combination of pembrolizumab (PD-1 inhibitor) and enfortumab vedotin (antibody-drug conjugate), establishing it as the new standard of care for locally advanced and metastatic urothelial carcinoma. However, the number of patients with the plasmacytoid subtype included in this trial is unknown and no sub-analysis was performed (17). To further clarify this issue, there is currently an ongoing phase II study using pembrolizumab and enfortumab vedotin in treatment of metastatic bladder cancer in patients with histologic subtypes, but no preliminary data is available (NCT05756569) (18).

To our knowledge, there are no existing reports of patients with PUC treated with the pembrolizumab and enfortumab vedotin. Here, we present three patients with stage IV PUC treated with this combination.

## 2 Case presentations

### 2.1 Patient 1

**Figure 1:**
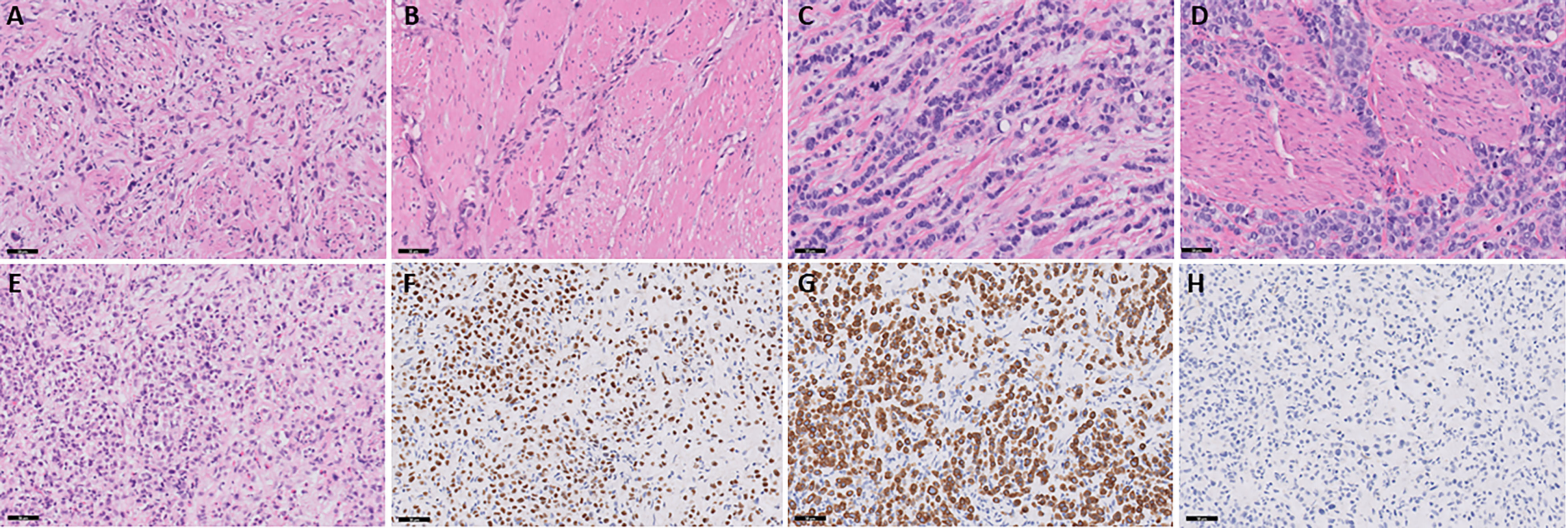
Slides **(A, B)** represent patient 1. **(A)** Muscle-invasive plasmacytoid urothelial carcinoma. Discohesive single individual tumor cells are seen diffusely infiltrating around and through muscular fascicles. The tumor cells display eccentric nuclei with peripheral eosinophilic cytoplasm, H&E stain, 20x magnification. **(B)** Large muscular fascicles are seen with tumor cells infiltrating through fascial planes, which is commonly seen in plasmacytoid urothelial carcinoma. The tumor cells show eccentric nuclei with peripheral eosinophilic cytoplasm, as well as signet ring cell forms, which are characteristic of plasmacytoid urothelial carcinoma, H&E stain, 20x magnification. Slides C and D represent patient 2. **(C)** Muscle-invasive plasmacytoid urothelial carcinoma. Tumor cells are seen in single file lines and are diffusely infiltrative through a myxoid stroma. Note the presence of an atypical mitotic figure, as well as scattered plasmacytoid and signet ring cell forms, H&E stain, 20x magnification. **(D)** Note the infiltration through and around muscular fascicles, as well as the abundant mitotic figures, H&E stain, 20x magnification. Slides E-H represent patient 3. **(E)** Invasive plasmacytoid urothelial carcinoma involving the lamina propria. The sample is superficial sample with no muscularis propria identified; however, the tumor is diffusely infiltrating through lamina propria with many discohesive cells with eccentric nuclei and peripheral eosinophilic cytoplasm, H&E stain, 20x magnification. **(F)** Nuclear staining is seen with the GATA-3 immunostain, GATA-3 stain, 20x magnification. **(G)** Membranous and cytoplasmic staining is seen with the CK7 immunostain, CK7 stain, 20x magnification. **(H)** E-cadherin staining is lost in the tumor cells, which is common in plasmacytoid urothelial carcinoma, E-cadherin stain, 20x magnification.

Patient 1 is a 68-year-old male with a medical history of coronary artery disease, chronic kidney disease, type 2 diabetes, and hypertension who presented with right-sided hydronephrosis following three months of gross hematuria, which required blood transfusion. A CT scan performed three weeks prior at an outside hospital revealed a 3.6 cm x 2 cm right-sided bladder mass. He initially underwent a transurethral resection of the bladder tumor (TURBT) with right ureteral stent placement. Pathology confirmed muscle-invasive high-grade urothelial carcinoma, plasmacytoid subtype with >75% of the tumor showing PUC features (**Figure 1**) with a low tumor mutational burden of 6 mutations per Mb. Staging with FDG PET-CT scan revealed metastatic right iliac chain and lower aortocaval lymph nodes.

**Figure 2:**
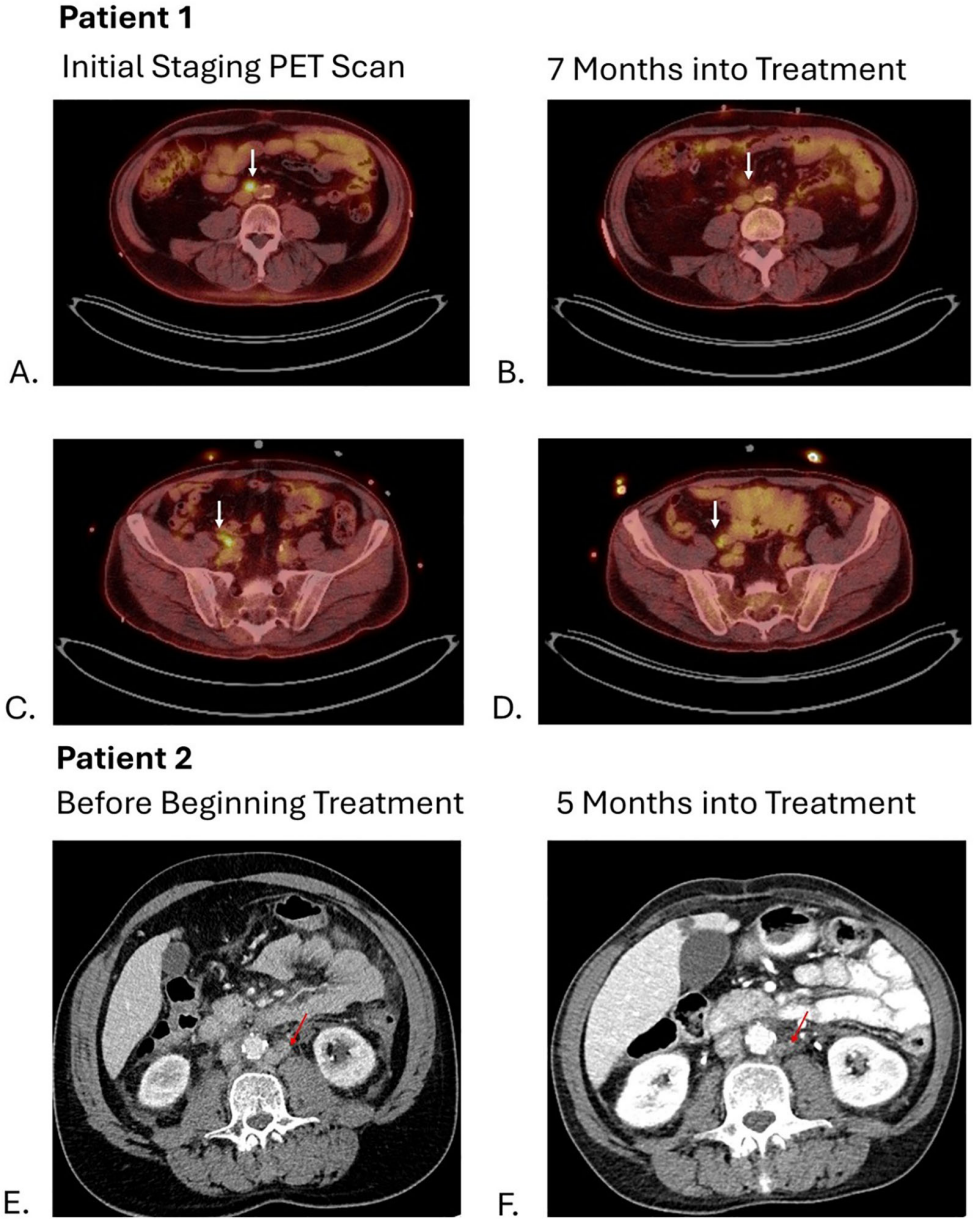
Arrows on images **(A, C)** show hypermetabolic activity of aortocaval and right common iliac lymph nodes respectively with decrease in metabolic activity shown 7 months into treatment on images **(B, D)**. Arrows on images **(E, F)** show decrease in size of left para-aortic lymph nodes from 11 mm to 3 mm.

The patient started treatment with pembrolizumab/enfortumab vedotin two months after the diagnosis. Following cycle 1, the patient developed a grade 2 rash—presumed to be related to enfortumab vedotin—which resolved with corticosteroid therapy. Subsequently, his enfortumab vedotin dose was reduced by 20% (1 mg/kg) and the rash did not recur. A follow-up PET scan at three months showed a partial response, with resolution of the right iliac chain lymphadenopathy and decreased metabolic activity in the aortocaval lymph nodes. However, his treatment was delayed by two months due to multiple hospitalizations from pyelonephritis, heart failure exacerbations, and flash pulmonary edema attributable to his pre-existing comorbidities. At seven months into treatment, a PET scan demonstrated stable disease compared to his previous scan (**Figure 2**), and the patient is now eight months into treatment on cycle 11 without evidence of progression with an overall a partial response to pembrolizumab/enfortumab vedotin.

### 2.2 Patient 2

Patient 2 is a 67-year-old male with a 45-pack-year smoking history who presented with gross hematuria. A CT scan revealed a thick-walled urinary bladder with urothelial enhancement, along with retroperitoneal and mediastinal adenopathy. He underwent TURBT and pathology revealed muscle invasive high-grade urothelial carcinoma, plasmacytoid subtype with >80% of the tumor showing PUC features (**Figure 1**). The tumor was microsatellite stable but showed a high tumor mutational burden of 32 mutations per Mb. A staging PET scan demonstrated multiple hypermetabolic and enlarged portacaval and retroperitoneal lymph nodes.

The patient was initiated on treatment with pembrolizumab/enfortumab vedotin. During cycle 4, he developed grade III immunotherapy-related colitis, requiring hospitalization and a prolonged steroid taper.

A follow-up CT of the abdomen and pelvis performed five months (**Figure 2**) into treatment revealed stable disease with further decrease in size of the abdominal and pelvic lymph nodes. As a result, patient resumed treatment with enfortumab vedotin and pembrolizumab combined with infliximab with no additional flares of colitis.

### 2.3 Patient 3

Patient 3 is a 43-year-old male with a past medical history of HIV, neurosyphilis, and hypertension that presented originally with nocturnal enuresis and bilateral lower extremity swelling. One month later, he was admitted to the hospital for sepsis secondary to a urinary tract infection (UTI). Cystoscopy showed a diffusely irregular bladder, particularly thickened along the trigone, with no discrete exophytic tumors visualized. Multiple biopsies from the bilateral ureteral orifices, bladder trigone, bladder neck, and prostate confirmed high-grade urothelial carcinoma, plasmacytoid subtype with nearly the entire tumor showing PUC features. The tumor samples were superficial and without muscularis propria but invasion into the lamina propria was identified (**Figure 1**) with a low tumor mutational burden of 5 mutations per mb. A staging FDG-PET scan revealed multiple hypermetabolic and enlarged portacaval and retroperitoneal lymph nodes concerning for metastatic involvement, along with innumerable small sclerotic lesions throughout the axial and appendicular skeleton, consistent with osseous metastases. Patient 3 was started on pembrolizumab/enfortumab vedotin, though his first initial two-week infusion was delayed due to hospitalization for pneumonia and a UTI. As skeletal metastases were present, the patient started Zometa infusion every three months. Treatment was complicated by multiple febrile UTIs, anemia, and difficulty tolerating infusions due to symptoms of dizziness, fatigue, weakness, and decreased appetite. These issues led to repeated delays during cycle 1 and cycle 2, ultimately necessitating a 20% dose reduction of enfortumab vedotin. Cycle 3 was further delayed due to anemia requiring blood transfusions. A CT scan of the abdomen and pelvis before cycle 3 revealed stable disease compared to the initial PET scan. Subsequent CT chest and CT urogram before cycle 5 also demonstrated no evidence of disease progression.

Following the completion of cycle 6, the patient was hospitalized with what was suspected to be a viral meningitis. One week later, he was re-admitted due seizures and cytology from the CSF revealed leptomeningeal carcinomatosis. He was transitioned to hospice care and passed away eight months after his initial diagnosis.

## 3 Discussion

PUC is a rare and aggressive histological subtype of urothelial carcinoma. In a retrospective cohort study of 49 patients with PUC, the survival time was 15 months among those with pT4 disease (19). A meta-analysis found that overall survival in PUC was worse than in conventional urothelial carcinoma with borderline significance after adjusting for other clinicopathological characteristics (3). Additionally, a retrospective study of 98 patients reported a median overall survival of 8 years for conventional urothelial carcinoma compared to 3.8 years for PUC (1).

The same meta-analysis found that PUC was more likely to present at stage pT3 (OR 3.84, p=0.0002) and had a significantly higher likelihood of ureteral margin positivity (OR 12.18, p < 0.00001), which may contribute to its aggressive nature (3). PUC also has a high propensity for metastasis, with a 20-year retrospective study of 56 patients showing a median time to metastasis of just 6.5 months (8). The most common site of metastasis in this study was the peritoneum (45.2%) followed by the bowel (26.2%), bone (26.2%), lymph nodes (21.4%) and liver (11.9%) (8).

A key factor driving the rapid metastatic spread of PUC is the frequent loss of E-cadherin, a cell adhesion molecule (3, 5, 6, 8). In a genomic analysis, CDH1 alterations -the gene encoding E-cadherin – were found in 61% of cases (10). The loss of E-cadherin causes the discohesive nature of cancer cells, facilitating invasion into surrounding tissue and lymphatic structures (9). This was evident in the case of one patient (Patient 3), whose tumor exhibited complete loss of E-cadherin expression (**Figure 2**), and who presented with widespread lymph node and osseous metastatic disease.

Historically, PUC has been treated similarly to conventional urothelial carcinoma using chemotherapy. However, PUC exhibits notoriously low chemo sensitivity (7, 9, 12, 13). In an analysis of 64 PUC patients and 418 with conventional urothelial carcinoma patients, the ypT0N0 rates after neoadjuvant chemotherapy (NAC) was significantly lower in PUC (10% *vs*. 33% (p=0.03) (7). Additionally, PUC was associated with lack of tumor downstaging and positive surgical margins at the time of radical cystectomy. Only 10% of PUC patients achieved complete response with NAC, while another study reported a complete response rate of only 12% (10).

Given the poor response to chemotherapy, immunotherapy has emerged as promising alternative for PUC. The KEYNOTE-045 trial demonstrated superior median 1-year overall survival with pembrolizumab compared to chemotherapy at 44.2% and 29.8% respectively in urothelial carcinoma (9). The response rate to immune checkpoint inhibitors (ICIs) for urothelial carcinoma previously has been reported at 38% (10). Interestingly, PUC is not typically associated with high PD-L1 expression, with only 5% of PUC patients in this study exhibited expression of PD-L1. However, PD-L1 expression is not always predictive of response of ICI (6, 10, 20).

Case reports have shown variable responses to ICIs as seen in **Table 1**. While one case reported progression on pembrolizumab (21), three other case studies reported favorable responses (5, 12, 22), and a larger study of 19 PUC patients found 32% complete radiographic response to ICIs (10).

**Table 1:** Previous case reports of plasmacytoid urothelial carcinoma (PUC) treated with immune checkpoint inhibitor therapy.

Case	Treatment	Outcome
61 year-old female with PUC with disease progression on cisplatin	Pembrolizumab	No evidence of recurrence or progression after neoadjuvant and adjuvant pembrolizumab one year post surgery (12)
70 year-old cisplatin-ineligible male with PUC	Pembrolizumab	Partial response after cycle 4. Increase in size of one mass after cycle 9 and otherwise stable. Continuing treatment (22)
19 PUC patients	Anti-PD1/PDL1 monotherapy	32% had complete radiographic response (10)
75 year-old female with PUC treated with gemcitabine/cisplatin	Pembrolizumab	Cancer progression and eventual death (21)
75 year-old male with PUC and initial complete response to neoadjuvant paclitaxel, gemcitabine, and cisplatin with development of metastatic disease to rectum	Dual HER1 targeting therapy and subsequently enfortumab vedotin	Complete response to enfortumab vedotin (5)

In addition to pembrolizumab, enfortumab vedotin has shown a survival benefit in the treatment of urothelial carcinoma. Enfortumab vedotin is an antibody-drug conjugate that targets nectin-4, a poliovirus receptor-altered protein that mediates calcium independent cell-cell adhesions (23). It remains unclear whether the nectin-4 positivity correlates with the response to enfortumab vedotin (23). However, a pre-clinical study shows that the downregulation of nectin-4 induces resistance to enfortumab vedotin, suggesting some degree of nectin-4 expression is necessary for enfortumab vedotin sensitivity (24). Notably, the plasmacytoid variant of urothelial carcinoma has been shown to have some of the highest expression of nectin-4 with 88.9% of PUC samples staining positive in one study (25).

Enfortumab vedotin was first granted accelerated approval after the high response rate of 43% was shown in patients with nectin-4 expressing tumors in the EV101 trial (11). When the pembrolizumab/enfortumab vedotin combination showed an objective response rate of 68% in cisplatin-ineligible urothelial carcinoma patients, the FDA granted accelerated approval (26). Recently, the updated EV302 trial displayed a survival benefit with the pembrolizumab/enfortumab vedotin group having a median overall survival of 33.8 months compared to 15.9 month in the chemotherapy group (HR 0.51). This improvement led to full FDA approval of the combination in patients with urothelial carcinoma, establishing it as the standard of care (17). However, the trial included only 0.9% of patients with urothelial carcinoma subtypes and 11.3% mixed histology (divergent differentiation), meaning the benefit in PUC remains unknown (17). Currently, only one case study has examined enfortumab vedotin in a PUC patient which resulted in a complete response (5).

In our study, three patients were treated with the pembrolizumab/enfortumab vedotin combination. Two patients experienced side effects, one developed a rash that resolved with steroids, while the other had immunotherapy-related colitis controlled with steroids and infliximab. He did well after pembrolizumab was resumed. Two out of three patients had achieved partial response and their treatment is ongoing. Unfortunately, one patient had progression after 5 cycles, and his treatment was interrupted due to multiple hospitalizations with patient ultimately passing away due to leptomeningeal dissemination. Patient 3 had significant co-morbidities with significant treatment delays due to treatment related side effects. This could have plausibly negatively influenced treatment efficacy compared to the other two patients who faced less significant delays.

Patient 2 was the only patient to have a high tumor mutational burden which has traditionally been shown to be related to a positive response to immunotherapy. Patient 1, however, was able to also maintain a stable response on the therapy regimen with a low tumor mutational burden, and patient 3 succumbed to the disease also with a low mutational burden. Nectin-4 expression was not obtained in these three patients. As it has been shown to relate to response to enfortumab vedotin as noted above, this is limitation of this case series and a key area of future investigation. Additionally, PD-L1 expression levels were only obtained for patient 1 which showed PD-L1 positivity (CPS 100) and E-cadherin expression only obtained for patient 3. This is an additional limitation of this study and an area that could be investigated further to determine the prognostic and predictive values of these markers.

Despite the recent advances in treatment options for urothelial carcinoma as demonstrated in the EV302 trial, more options are needed for the histologic subtypes of urothelial carcinoma (15). Currently, there are three on-going clinical trials (NCT04624399, NCT06417190, and NCT03866382) investigating treatments specifically for histologic subtypes with two additional trials looking at metastatic GU tumors (NCT03744793 and NCT02496208) as summarized below in **Table 2**. The ABACUS-2 trial looked at the efficacy of neoadjuvant atezolizumab in patients with muscle invasive, non-conventional urothelial carcinoma of the bladder. Among the 33 patients who completed treatment, central pathology review discrepancies limited the final analysis to 24 patients, with a complete pathologic response observed in 38% (27). These findings highlight the need for further therapeutic options tailored to patients with histological subtypes of urothelial carcinoma.

**Table 2:** Clinical trials involving treatment of urothelial carcinoma.

Trial	Phase	Objective	Eligibility	Status	Enrollment
NCT04624399	Phase II	Evaluate the safety and efficacy of neoadjuvant atezolizumab in patients with non-conventional urothelial, muscle invasive bladder cancer	Cisplatin-ineligible, fit for cystectomy, residual disease after TURBT, mixed or pure non-conventional urothelial carcinoma subtype	Re-opened trial, recruiting	58 participants targeted
NCT06417190	Phase II	Evaluate the outcomes in patients with muscle invasive bladder cancer with a histological subtype who receive neoadjuvant chemotherapy with or without immunotherapy followed by trimodal therapy	Diagnosis of muscle invasive bladder cancer with a histological subtype	Recruiting	20 participants targeted
NCT03866382	Phase II	Evaluate efficacy of cabozantinib in combination with nivolumab and ipilimumab in treating patients with metastatic GU tumors with a histological subtype	Metastatic genitourinary urinary cancer of a histological subtype	Recruiting	314 participants targeted
NCT03744793	Phase II	Evaluate the response rate in patients with methylthioadenosine phosphorylase (MTAP)-deficient metastatic urothelial cancer treated sequentially with pemetrexed and avelumab	MTAP-deficient metastatic urothelial carcinoma	Active	18 participants
NCT02496208	Phase I	Determine the dose limiting toxicity and recommended dosing of cabozantinib s-malate and nivolumab with or without ipilimumab in patients with metastatic genitourinary tumors	Metastatic genitourinary urinary tumors that have progressed to at least one stage on standard treatment or no standard treatment available to prolong survival	Active	152 participants

## 4 Patient perspectives

### 4.1 Patient 1

Receiving the diagnosis of bladder cancer was overwhelming, especially with my existing health conditions. The months leading up to my diagnosis were difficult—I had persistent blood in my urine, fatigue, and needed blood transfusions. When I learned that the cancer had spread to my lymph nodes, I was afraid of what that meant for my future. Starting treatment with pembrolizumab and enfortumab vedotin gave me hope, but it hasn’t been without challenges. The rash after my first cycle was frustrating, though it improved with medication. More concerning were the infections and hospitalizations that delayed my treatment; each setback made me worry about whether the cancer would progress.

Emotionally, this journey has been a rollercoaster. I have moments of gratitude when scans show improvement, but also anxiety when faced with complications. My daily life has changed—I have less energy, and my frequent medical visits make it hard to maintain independence. Despite these difficulties, I remain hopeful. The treatment is keeping my cancer stable, and I focus on taking things one step at a time.

### 4.2 Patient 2

When I first saw blood in my urine, I knew something wasn’t right, but I never expected to hear the words “bladder cancer.” The news was shocking, and the thought of it spreading to my lymph nodes was terrifying. I’ve been a smoker for most of my life, and part of me feared this day would come. After my diagnosis, everything moved quickly—scans, surgery, and then starting treatment with pembrolizumab and enfortumab vedotin.

I later learned that my cancer was a rare and aggressive type called plasmacytoid urothelial carcinoma, which made my situation even more daunting. My doctors told me that treatment options were limited, which was difficult to hear. Still, I wanted to fight for as much time as possible.

The first few cycles of treatment went smoothly, but then I developed severe diarrhea that landed me in the hospital. The colitis was one of the hardest parts of this journey. I was exhausted, losing weight, and worried I might have to stop treatment altogether. Thankfully, with the right medications and a slow steroid taper, I was able to recover. Now, I’m back on treatment with an added medication to prevent the colitis from returning.

Even though the side effects have been challenging, they’ve been tolerable, and my latest scans show stable disease. I know this cancer is aggressive, but I’m grateful to still be here. This experience has completely changed my life—I’ve had to adjust my daily routine, deal with uncertainty, and learn to live with the ups and downs of treatment. But every day I wake up is a blessing, and I hold on to the progress I’ve made.

## 5 Conclusion

We presented three cases of patients with the plasmacytoid histologic subtype of urothelial carcinoma. Two out of three patients have stable/partial disease with manageable side effects while receiving the combination of pembrolizumab/enfortumab vedotin, supporting its potential as a viable treatment option for this aggressive variant.

## Data Availability

The original contributions presented in the study are included in the article/supplementary material. Further inquiries can be directed to the corresponding author.
